# Smoking and the risk of diabetic nephropathy in patients with type 1 and type 2 diabetes: a meta-analysis of observational studies

**DOI:** 10.18632/oncotarget.21478

**Published:** 2017-10-04

**Authors:** Ning Jiang, Feng Huang, Xiurong Zhang

**Affiliations:** ^1^ Department of Traditional Chinese Medicine, Maternal and Child Health Care of Shandong Province, Key Laboratory of Birth Regulation and Control Technology of National Health Family Planning Commission of China, Jinan, Shandong Province, China; ^2^ Department of Orthopedics, Shandong Provincial Hospital of the Chinese People’s Armed Police Forces, Jinan, Shandong Province, China

**Keywords:** diabetic nephropathy, end stage renal disease, macroalbuminuria, microalbuminuria, cigarette smoking

## Abstract

**Background:**

Conflicting evidence exists for observational studies on whether tobacco smoking is a risk factor for diabetic nephropathy (DN) in patients with type 1 (T1DM) and type 2 diabetes mellitus (T2DM). In this meta-analysis, we aimed to assess the effects of tobacco smoking on the development of DN.

**Materials and Methods:**

We searched MEDLINE and EMBASE databases from their inception to March 31^st^, 2017 for cross-sectional, case-control, and prospective cohort studies. We screened reference lists of retrieved articles. Summary relative risks (SRRs) and 95% confidence intervals (CIs) were calculated using a random-effects model.

**Results:**

A total of nineteen observational studies (1 case-control, 8 cross-sectional and 10 prospective cohort studies) were identified, involving more than 78,000 participants and a total of 17,832 DN cases. Compared with never-smokers, there was an augmented SRR (95% CI) of DN in ever-smokers in patients with T1DM (1.31 [1.06–1.62]; *P* = 0.006) and T2DM (1.44 [1.24–1.67]; *P* < 0.001), respectively. In patients with T1DM, the SRR (95% CI) was 1.25 (0.86–1.83) for microalbuminuria only, 1.27 (1.10–1.48) for macroalbuminuria only, and 1.06 (0.97–1.15) for end-stage renal disease (ESRD). In patients with T2DM, the SRR (95% CI) associated with ever smoking was 1.46 (0.94–2.26) for microalbuminuria only, 1.72 (1.04–2.84) for macroalbuminuria only, and 1.10 (0.36–3.33) for ESRD.

**Conclusions:**

Our meta-analysis suggests evidence for cigarette smoking as an independent risk factor for the development of DN in patients with both T1DM and T2DM.

## INTRODUCTION

The incidence of diabetes mellitus (DM) is increasing worldwide and not only does it affect quality of life for these patients but the condition also results in a huge economic burden for individuals and countries [1]. Diabetic nephropathy (DN) is one of the most common and severe microvascular complications of DM and is the leading cause of end-stage renal disease (ESRD) [2]. Once those with diabetes develop DN, pathological changes often develop progressively and cannot be easily reversed [3, 4]. DN is characterized by persistent albuminuria, arterial blood pressure elevation, and a decline in the glomerular filtration rate (GFR) [5]. Established risk factors for the development of DN include genetic susceptibility, advanced age, male gender, hypertension, poor glycemic control, duration of diabetes, and an unfavorable lipid profile [6, 7].

Cigarette smoking is undoubtedly one of the leading causes of premature death, predominantly resulting in cardiovascular disease and cancer [8]. With regard to the deleterious effect of smoking on the kidneys, data from a recent systematic review and meta-analysis of prospective studies suggested that there is an independent association between smoking and incident chronic kidney disease (CKD) in the general population [9]. In relation to DN, smoking is considered to have harmful effects contributing to the development and progression of DN [5, 10]. However, data supporting this statement have been inconsistent. To the best of our knowledge, no systematic reviews or meta-analyses have assessed smoking as a risk factor for the development of DN, although there are many observational studies on this issue [1, 5, 10–25]. Therefore, to better characterize the association between tobacco smoking status and the development of DN in patients with type 1 (T1DM) and type 2 diabetes (T2DM), we conducted a comprehensive meta-analysis of observational studies. This meta-analysis followed the guideline on meta-analysis of observational studies in epidemiology (MOOSE) [26].

## MATERIALS AND METHODS

### Literature search

Two investigators (HF and JN) independently screened the original articles, which were written in English. We comprehensively searched for relevant studies in two databases (EMBASE, http://www.embase.com/) and MEDLINE (PubMed, http://www.ncbi.nlm.nih.gov/pubmed/) from the inception of these databases to March 31^st^, 2017. The search terms included the following key words: 1) smoking OR cigarette OR tobacco OR nicotine; 2) proteinuria diabetes OR diabetic macroalbuminuria OR diabetic microalbuminuria OR diabetic nephropathy OR diabetic nephropathies; and 3) cohort OR case-control OR cross-sectional. The references of included studies and relevant reviews were also checked for eligible studies.

### Exposure and outcome measures

Some articles reported on individuals ever smoking, and several included data for individuals who had formerly smoked or were currently smoking, therefore results on ever smoking were derived from combining the results for former and current users. Current users were considered to be ever users in some studies that only reported current smoking habits.

Given proteinuria is known as the main clinical manifestation of DN, the outcome of interest was the urine albumin-to-creatinine ratio (UACR) or urine albumin excretion rates (UAER). According to the UACR or UAER, DM patients were divided into 3 groups: normoalbuminuria (UAER: < 30 mg/d or UACR: < 30 mg/g), microalbuminuria (UAER: 30–300 mg/d, UACR: 30–299 mg/g) and macroalbuminuria (UAER: ≥ 300 mg/d or UACR: ≥ 300 mg/g). Patients in the micro- and macroalbuminuria groups were considered to have DN.

### Study selection

Studies were included according to the following criteria: 1) the design was based on an observational study evaluating the association between smoking and the development of DN; 2) the risk estimates and their confidence intervals [CIs] were at least adjusted or matched for age and hypertension; 3) if more than 1 study originated from the same population, the latest or most informative study was included. Animal experiments, chemistry, cell line studies, editorials, commentaries, review articles, and case reports were all excluded. Data on other forms of tobacco use, such as cigar and pipe, were not analyzed. We did not consider the grey literature. Two investigators (HF and JN) independently reviewed all potentially relevant articles to determine whether an article met the general inclusion criteria, and disagreement was resolved by discussion between the two investigators.

### Data extraction

Two researchers (HF and JN) independently extracted the following data from each publication: the first author’s last name, year of publication, study locations, sample size, gender, age, smoking category, the methods of assessment of outcomes, duration of follow-up in cohort studies, relative risk [RR] and 95% CI, and co-variables that were adjusted for in the analysis. From each study, we extracted the risk estimates that reflected the greatest degree of control for potential confounders.

### Statistical analysis

Based on a random-effects model, we pooled study-specific RRs and their 95% CIs from all included studies. This random- effects model was developed by DerSimonian and Laird, which robustly considers heterogeneity among studies [27]. We used a fixed effects model to obtain overall combined estimates for DN risk for studies that reported results separately for different pack-years of smoking [16, 28], before and after diagnosis of diabetes [5], men and women [14] and whether there was different expression of the rs2295490 genotype [1]. For studies reporting risk estimates according to continuous measure of pack-years [10], we calculated the risk estimates according to the mean smoking dose.

In assessing heterogeneity among studies, we used the Cochran Q and I^2^ statistics. The *I*^2^ statistic is the percentage of total variation across studies due to heterogeneity rather than through chance. Results were defined as heterogeneous for *P*-values < 0.10 or *I*^2^ was > 50% [29]. We explored the origin of heterogeneity by subgroup analysis according to study characteristics such as study design, locations, confounders, outcome of DN (microalbuminuria, macroalbuminuria, and renal insufficiency). Heterogeneity was also evaluated by random-effects meta-regression, which used the method of maximum likelihood approaches, where appropriate. Sensitivity analysis was used to assess the effect of a single study on the overall pooled estimates by excluding each study in turn.

Publication bias was assessed using funnel plots and the further Begg’s adjusted rank correlation and Egger’ regression asymmetry tests [30, 31]. *P* < 0.10 was considered to be representative of a significant statistical publication bias. We also performed a nonparametric “trim and fill” procedure to further assess the possible effect of publication bias in our meta-analysis. All statistical analyses were performed using STATA, version 11.0 (STATA, College Station, TX, USA). A two-sided *P* value < 0.05 was considered to be statistically significant.

## RESULTS

### Search results and study characteristics

**Figure 1:**
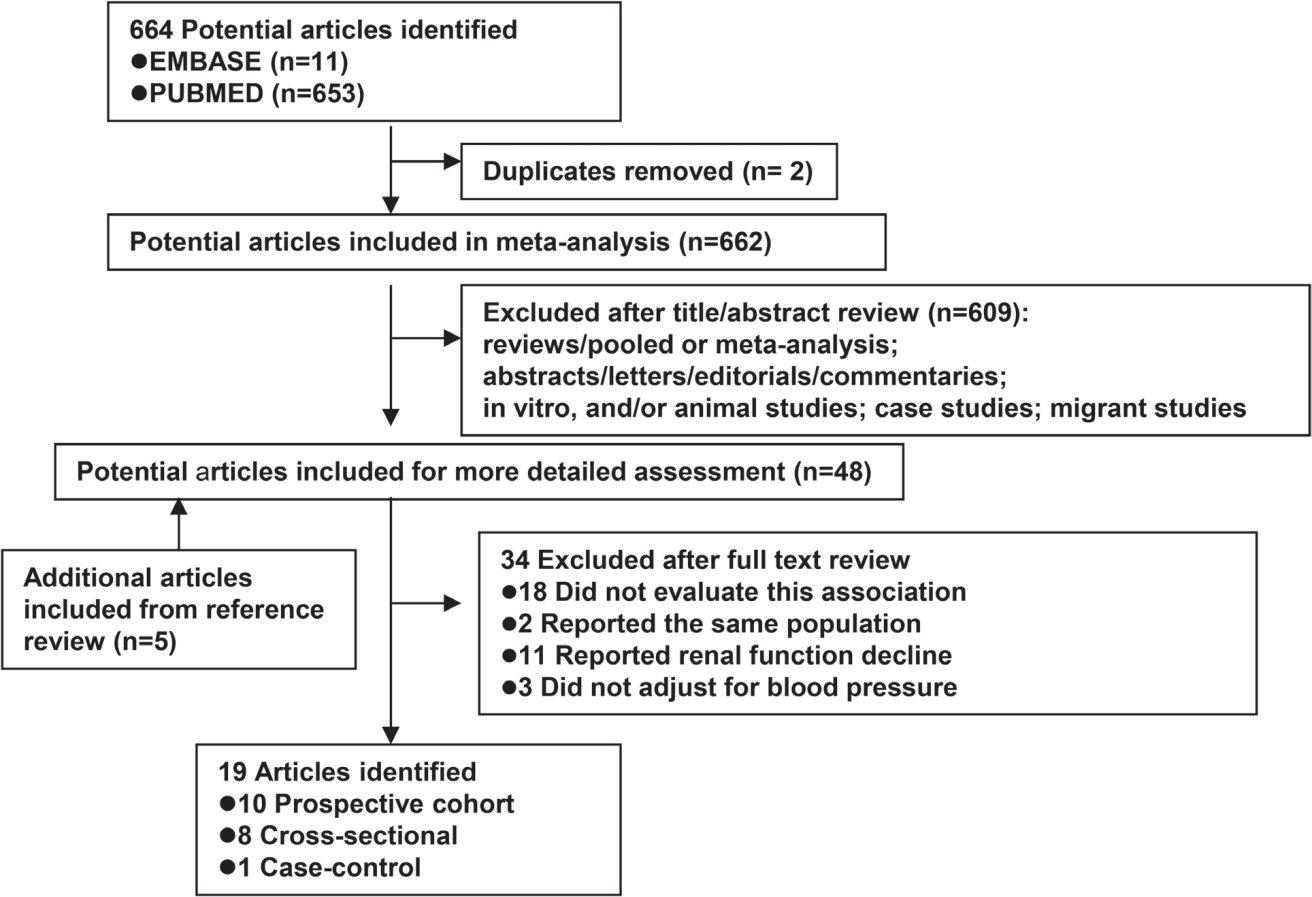
Flow diagram of systematic literature search on cigarette smoking and diabetic nephropathy in patient with type 1 and with type 2 diabetes

Based on the study selection criteria, we identified a total of 664 potentially relevant articles (653 articles from MEDLINE database and 11 articles from EMBASE database). Of the 664 individual papers, 48 papers were thoroughly assessed by reading their full-text. After reviewing the cross-reference list, a further 5 articles were identified. By studying these 53 articles, 34 were excluded for various reasons (Figure 1). Therefore, a total of nineteen articles were included in the meta-analysis; 10 prospective cohort, 1 case-control, and 8 cross-sectional studies (Supplementary Table 1). The continents or countries where the studies were conducted were: Asia (*n* = 8), the United States (*n* = 3), Europe (*n* = 7), and multi-centers (*n* = 1).

All articles were published between 1995 and 2016 and involved more than 78,000 participants and a total of 17,832 DN cases. In all studies, relative risk estimates were adjusted for age, gender, and blood pressure. All but one study made adjustments for duration of DM [11]. More than half of the included studies made adjustments for body mass index (BMI), HbA1c, and blood lipid levels. Some studies adjusted for hypertension and diabetes medications. Among the nineteen studies, only three focused on diabetic microalbuminuria [20, 21, 23], six focused on diabetic macroalbuminuria only [13, 14, 22, 24, 25, 28], four focused on micro-and macroalbuminuria combined [1, 15, 18, 19], two specifically focused on micro-, macroalbuminuria, and ESRD [10, 16], and two focused on ESRD [11, 12] or eGFR < 60 ml/min/1.73m^2^ [5, 17].

### Smoking and the development of DN in patients with T1DM

**Figure 2:**
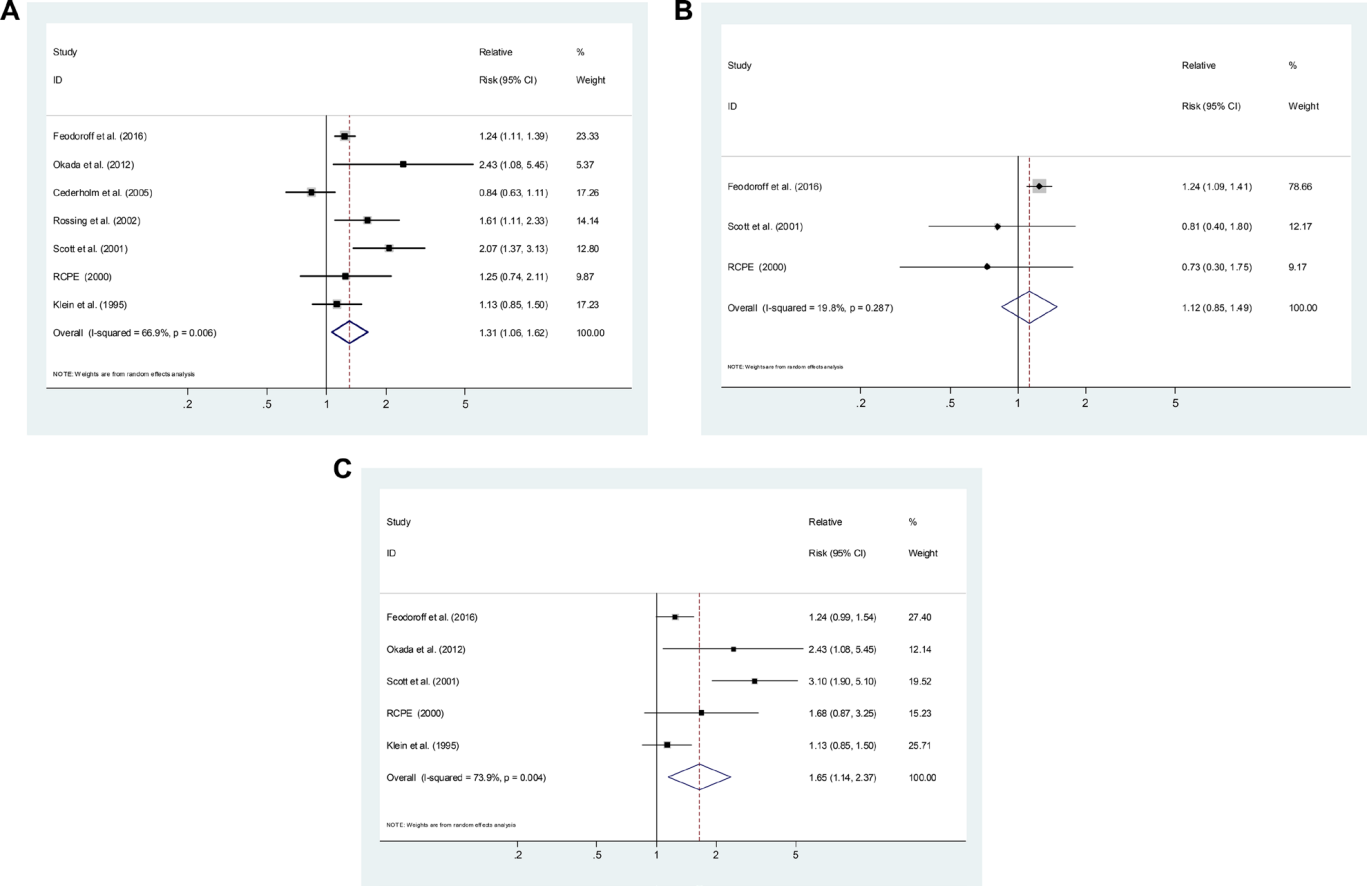
Estimates of the relative risk of developing diabetic nephropathy in patient with type 1 diabetes for (**A**) ever smokers, (**B**) former smokers, (**C**) current smokers.

Six cohort studies and 1 cross-sectional study [10, 14, 20, 22–24] reported risk estimates for the association between smoking and the development of DN in T1DM. Compared with never-smokers, ever-smokers were associated with an elevated risk of DN development (SRR = 1.31; 95% CI, 1.06–1.62), with evidence of heterogeneity (*P* = 0.006, I^2^ = 66.9%; Figure 2A). Three studies [10, 23, 24] presented results on former smoking, with a SRR of 1.12 (95% CI, 0.85–1.49; *P* = 0.287, I^2^ = 19.8%; Figure 2B). Five studies [10, 14, 22–24] presented results on current smoking, with a SRR of 1.65 (95% CI, 1.14–2.37; *P* = 0.004, I^2^ = 73.9%; Figure 2C). Current smokers had a significantly higher risk of DN development than former smokers (P for difference = 0.011).

As shown in Table 1, Subgroup analysis restricted to prospective cohorts led to a similar SRR of 1.26 (95% CI, 1.03–1.56; *P* = 0.009, I^2^ = 67.7%). Table 1 Subgroup analysis was performed by the outcome of DN: microalbuminuria only (SRR = 1.25, 95% CI, 0.86–1.83), macroalbuminuria only (SRR = 1.27, 95% CI, 1.10–1.48), ESRD (SRR = 1.06, 95% CI 0.97–1.15). Subgroup analysis was also performed by locations: Asia (SRR = 2.43; 95% CI, 1.08–5.45), Europe (SRR = 1.18; 95% CI, 0.92–1.52) and the USA (SRR = 1.50; 95% CI, 0.83–2.71). We also conducted a sensitivity analysis by omitting one study at a time and calculating the SRRs for the remainder of the studies, and found that there were no changes in the direction of effect when any one study was excluded (Supplementary Figure 1A).

**Table 1:** Stratified analyses for the association between ever smoking and diabetic nephropathy

Subgroup	T1DM				T2DM			
	No.	SRR (95% CI)	P_h_, I^2^ (%)	P_d_	No.	SRR (95% CI)	P_h_, I^2^ (%)	P_d_
	7	1.31 (1.06–1.62)	0.006, 66.9		14	1.44 (1.24–1.67)	< 0.001, 71.6	
**Design**				0.254				0.046
Prospective	6	1.26 (1.03–1.56)	0.009, 67.7		6	1.16 (1.01–1.34)	0.374, 6.7	
Cross-sectional/case-control	1	2.43 (1.08– 5.45)	-		8	1.67 (1.32–2.11)	< 0.001, 82.3	
**Locations**				0.897				0.169
Asian	1	2.43 (1.08–5.45)	-		7	1.62 (1.23–2.09)	0.068, 48.8	
European	4	1.18 (0.92–1.52)	0.031, 66.1		4	1.54 (1.13–2.09)	0.008,74.7	
USA	2	1.50 (0.83–2.71)	0.018, 82.1		2	1.19 (0.99–1.42)	0.564, 0	
**Outcome**								0.855
Microalbuminuria only	3	1.25 (0.86–1.83)	0.001, 84.7	0.754	3	1.46 (0.94–2.26)	0.018, 75.0	
Macroalbuminuria only	5	1.27 (1.10–1.48)	0.273, 22.3		4	1.72 (1.04–2.84)	0.007, 75.0	
ESRD	1	1.06 (0.97– 1.15)	-		2	1.10 (0.36–3.33)	0.052, 73.6	
**Adjustments by BMI**				0.868				0.414
Yes	6	1.33 (1.05–1.68)	0.003, 72.4		11	1.38 (1.17–1.63)	< 0.001, 70.3	
No	1	1.25 (0.74–2.11)	-		3	1.62 (1.36–1.92)	0.582, 0	
**Adjustments by Dyslipidemia**				0.991				0.187
Yes	3	1.25 (1.03–1.52)	0.216, 34.7		7	1.63 (1.27–2.09)	0.009, 64.8	
No	4	1.35 (0.88–2.08)	0.002, 80.1		7	1.30 (1.09–1.54)	0.008, 65.7	

### Smoking and the development of DN in patients with T2DM

**Figure 3:**
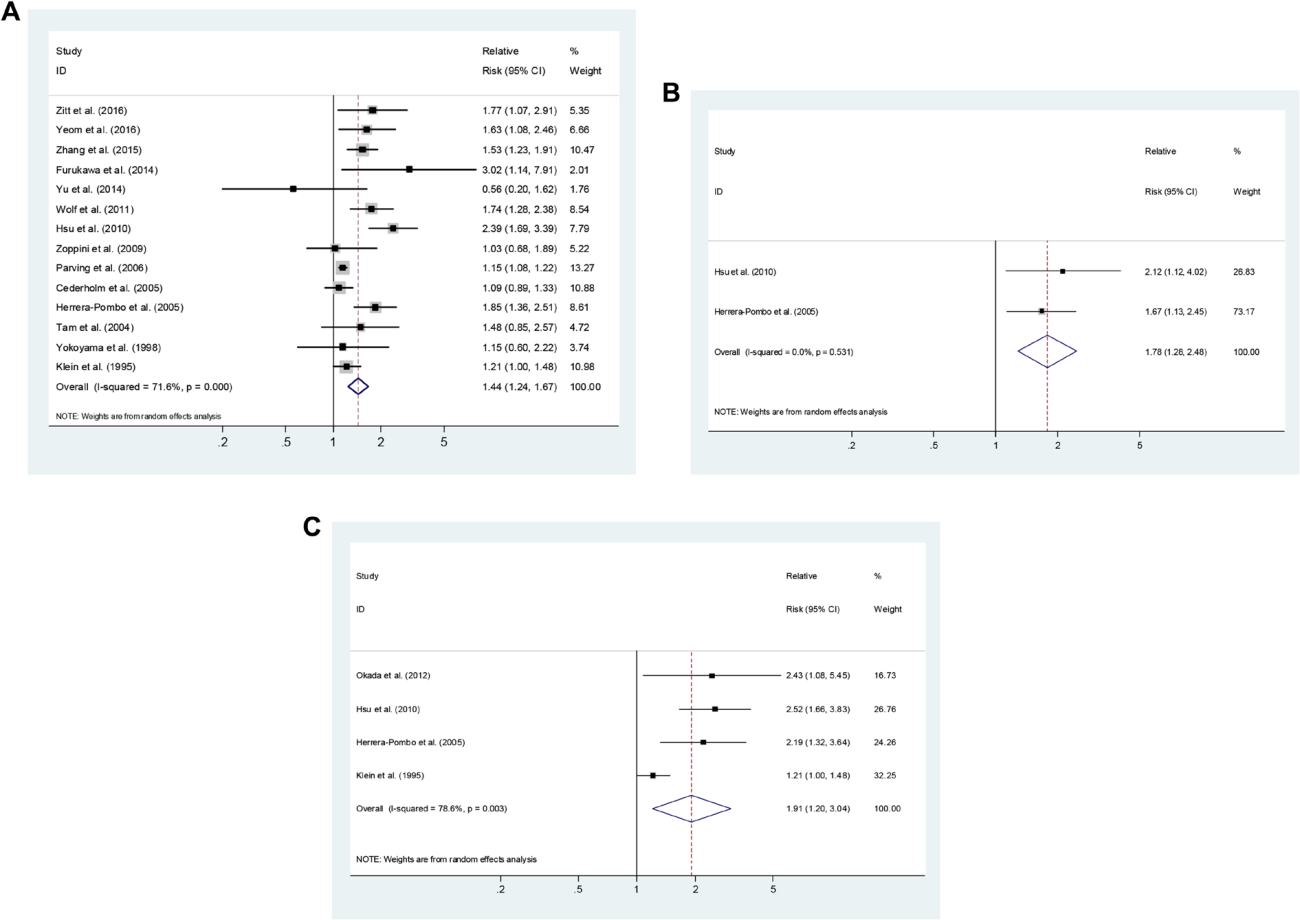
Estimates of the relative risk of developing diabetic nephropathy in patient with type 2 diabetes for **(A)** ever smokers, **(B)** former smokers, **(C)** current smokers.

Six cohort studies, 1 case-control study and seven cross-sectional studies [1, 5, 11–13, 15–21, 25, 28] reported risk estimates for an association between smoking and the development of DN in patients with T2DM. Compared with never-smokers, ever-smokers were associated with an elevated risk of DN development (SRR = 1.44; 95% CI, 1.24–1.67), with evidence of heterogeneity (*P* < 0.001, I^2^ = 71.6%; Figure 3A). One study [16] presented results on former smoking, with a RR of 2.12 (95% CI, 1.12–4.02). Four studies [14, 16, 19, 28] presented results on current smoking, with a SRR of 1.85 (95% CI, 1.03–3.32; *P* = 0.003, I^2^ = 82.6%; Figure 3B).

As shown in Table 1, Subgroup analysis was restricted to prospective cohort (SRR = 1.16; 95% CI, 1.01–1.34; *P* = 0.374, I^2^ = 6.7%) and cross-sectional/case-control studies (SRR = 1.67; 95% CI, 1.32–2.11; *P* < 0.001, I^2^ = 82.3%). Subgroup analysis was performed by the outcome of DN: microalbuminuria only (SRR = 1.46, 95% CI, 0.94–2.26), macroalbuminuria only (SRR = 1.72, 95% CI, 1.04–2.84), and ESRD (SRR = 1.10, 95% CI, 0.36–3.33), and by locations: Asia (SRR = 1.62; 95% CI, 1.23–2.09), Europe (SRR = 1.54; 95% CI, 1.13–2.09) and the USA (SRR = 1.19; 95% CI, 0.99–1.42). We also conducted a sensitivity analysis by omitting one study at a time and calculating the SRRs for the remainder of studies, and found that there were no changes in the direction of effect when any one study was excluded (Supplementary Figure 1B).

### Publication bias

**Figure 4:**
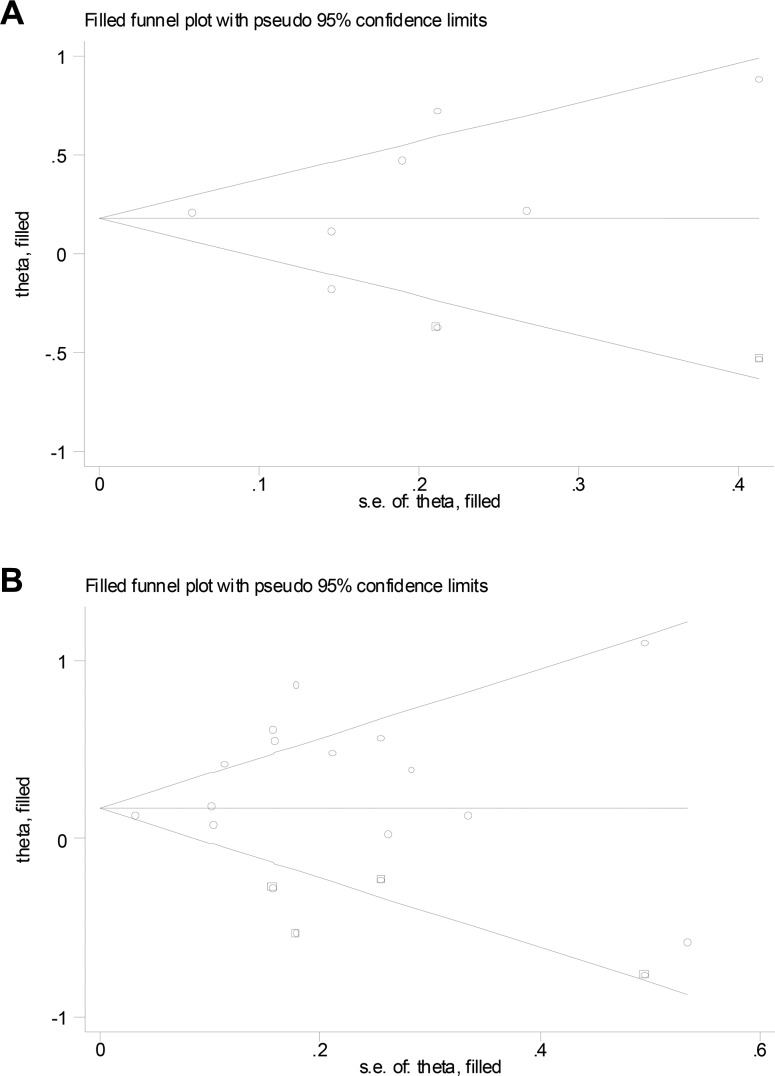
Filled funnel plot of log relative risk vs. standard error of log relative risks in studies that evaluated the effect of ever smoking on the development of diabetic nephropathy in patient with **(A)** type 1 and with **(B)** type 2 diabetes.

For studies on T1DM, Begg’s (*P* = 0.133) and Egger’s (*P* = 0.466) tests did not reveal evidence of publication bias but visual inspection of the funnel plots revealed significant asymmetry. The trim-and-fill method suggested that 2 additional risk estimates were needed to balance the funnel plot and the summary risk estimates lost statistical significance (SRR = 1.18; 95% CI, 0.95–1.47; Figure 4A). For studies on T2DM, Begg’s (*P* = 0.913) test did not reveal evidence of publication bias but visual inspection of the funnel plots and Egger’s test (*P* = 0.087) revealed significant bias. The trim-and-fill method suggested that 4 additional risk estimates were needed to balance the funnel plot and the summary risk estimates were still statistically significant, albeit much weaker (SRR = 1.24; 95% CI, 1.06–1.45; Figure 4B).

## DISCUSSION

Based on the current meta-analysis of ten prospective cohort, 8 cross-sectional, and 1 case-control studies, we found that ever smoking may be associated with an elevated risk of diabetic nephropathy in patients with type 1 and with type 2 DM. We believe these studies were well-organized. These associations were independent of major risk factors for nephropathy such as hypertension, DM duration, BMI, and dyslipidemia.

Although the exact mechanism is unknown, a possible pathological pathway of cigarette smoking resulting in DN has been proposed. First, smoking increases the concentration of carboxyhemoglobin, prothrombotic factors, and platelet activation [32], all of which can cause proinflammation, oxidative stress, and endothelial cell dysfunction (diminished nitric oxide availability and intimal cell hyperplasia), glomerulosclerosis, and tubular atrophy [33, 34]. In the literature, smokers with DM had thicker glomerular basement membranes compared with nonsmokers with DM [35]. *In vivo* studies have suggested that nicotine can promote mesangial cell proliferation and increase production of the extracellular matrix [36, 37]. The second putative pathway for smoking to result in DN has been linked to worse glycemic control and a less favorable lipid profile in patients with diabetes [10], both of which are independently associated with progression of DN [38]. The third mechanism is that smokers are unaware of the progression of a chronic disease (e.g., diabetes and hypertension) [16, 39–41], leading to a delay in diagnosis and treatment of their diseases, increasing the likelihood of complications, such as DN.

Recently, a systematic review and meta-analysis identified tobacco smoking as the significant risk factor for incidence and progression of CKD in the general population [9]. In addition, a positive association between cigarette smoking and the progression of CKD in IgA nephropathy [42] and idiopathic membranous nephropathy [43] has also been demonstrated in prospective studies. However, this relationship in patients with T1DM and T2DM remains unclear. A study from the USA showed that current smokers with T1DM have a higher risk of microalbuminuria (RR = 3.1, 95% CI, 1.9–5.1) during a 4-year follow-up period [23]. However, two other Danish prospective studies showed contradictory results: smoking was not associated with a decline in kidney function or the progression of albuminuria, or the development of microalbuminuria [44, 45]. For patients with T2DM, a German study showed that although smoking is associated with a decline in kidney function, it did not increase the risk of proteinuria [46].

In the meta-analysis of T1DM, a stronger risk was evident in current smokers than in former smokers (RR = 1.65 vs. 1.12, P for difference < 0.001), suggesting that cessation of smoking significantly decreased the risk of the development of DN. Furthermore, the elevated risk of DN in former smokers lost statistical significance. A prospective study by Chuahirun et al. [47] and Phisitkul et al. [48]. reported that those who quit smoking with T2DM had lower rates of alb/cr change (P = 0.021) or eGFR decline (P < 0.001) than smokers with T2DM, respectively. In addition, findings from a Korean cross-sectional study [5] indicated that smoking cessation in male patients with T2DM could delay the progression of DN or slow the decrease of glomerular function. These data supported the contention that smoking cessation is an effective renoprotective intervention in patients with type 1 and type 2 DM.

In the meta-analysis of the effects of cigarette smoking specifically on type 1 and type 2 DN, we observed similar summary risk estimations (SRR = 1.31 vs. 1.44), suggesting that cigarette smoking may exert an equally detrimental influence on T1DM and T2DM. When we performed meta-analysis according to different stages of DN, we found somewhat stronger, albeit not significant, risk estimation for microalbuminuria and macroalbuminuria compared with ESRD. These results indicated that the influence of smoking begins in the early stages of diabetes. In addition, cigarette smoking is possibly a weaker risk factor in the development of ESRD compared with hypertension and duration of DM [16]. However, it is important to note that only a small number (< 5) of studies were included, which assessed different stages of DN.

To make our results more reliable, we made concerted efforts in several aspects of the study as follows: (1) this meta-analysis is the first quantitative review of smoking status and the development of DN, which included a comprehensive, systematic search of the literature using a broad search strategy to identify studies. (2) This meta-analysis included 10 prospective studies, which should have eliminated selection and recall bias. (3) Smoking has effects on many factors, including BMI, vascular disease, dyslipidemia, glycemic control and other important physiological function, and most of the included studies were adjusted for potential confounders. (4) We performed subgroup and meta-regression analysis to explore the source of heterogeneity. We suggest that study design might be one of the sources of heterogeneity.

However, our study has some limitations, which should be considered. First, many studies were based on a cross-sectional or case-control design, which does not allow for causal inference and can overestimate measures of association. When restricting to prospective cohort studies, a significant, albeit weaker association was found between cigarette smoking and the development of DN in patients with T1DM and T2DM, respectively. Second, as with all observational studies, residual confounding cannot be completely ruled out. For example, smokers tend to be associated with heavy alcohol consumption behaviors [49]. Dunkler et al. found that moderate alcohol users with T2DM had a significantly decreased CKD risk compared with non-alcohol consumers with T2DM [50]. However, only one included study adjusted for alcohol consumption [13]. Furthermore, smokers tend to be thinner on average and have a less favorable lipid profile than non-smokers [10, 51]. The majority of studies included in our meta-analysis adjusted for BMI or dyslipidemia. However, a meta-analysis is not able to resolve confounding factors that could be inherent in the included studies.

A third limitation of our study was the high heterogeneity across studies that were present, which may be related to the study design, number of cases, method of exposure measurement, definition of DN, and adjustment for confounders. The definition, classification of smokers, and the methods used for quantifying tobacco exposure are different, which may yield varied results with regard to the strength of the association. In addition, the diagnostic criteria for DN varied, with some studies categorizing UACR 30–300 mg/g as microalbuminuria, UACR > 300 mg/g as overt nephropathy, while other studies defined DN as UAER > 30 mg/24 h on any annual evaluation. Considerable heterogeneity could make the results less robust. The meta-analysis of cohort studies on T2DM showed little variability, whereas significant heterogeneity was observed among cross-sectional/case-control studies. The meta-regression analysis suggested that study design was the most significant factor (*P* = 0.046), accounting for 21.7% of the significant heterogeneity observed among studies.

A fourth limitation was that assessment of a dose–response relationship was a major criterion for determination of the causality of an association. Due to the absence of smoking dose information in the included studies, we could not perform a quantitative review of the dose–response relationship. Only five studies [12, 16, 17, 20, 24] considered the medications for hypertension and/or diabetes e.g., angiotensin-converting enzyme inhibitor (ACEI) or Angiotensin Receptor Blocker (ARB), which were found to play a role in the reduction of albuminuria [52].

Finally, publication bias is of a concern since the grey literature tends not to be published in MEDBASE or EMBASE databases. This bias may have led to an overestimation of the true association. Indeed, Egger’s test (*P* = 0.087) provided evidence for such bias for studies on T2DM. Further examination using the trim-and-fill method suggested 4 additional risk estimates were needed to balance the funnel plot, and the summary risk estimates were still statistically significant, albeit these were weaker (SRR =1.24).

In conclusion, results from our meta-analysis of observational studies clearly demonstrate an adverse impact of smoking on the development of DN in patients with T1DM and T2DM.

## SUPPLEMENTARY MATERIALS FIGURE AND TABLE




