# Supplementary material for: Smoking and the risk of diabetic nephropathy in patients with type 1 and type 2 diabetes: a meta-analysis of observational studies
Source: Oncotarget. 2017 Oct 4;8(54):93209–18. doi: 10.18632/oncotarget.21478 (PMC5696256; doi:10.18632/oncotarget.21478)
Supplement: Supplementary file 1 [file oncotarget-08-93209-s001.pdf]

Smoking and the risk of diabetic nephropathy in patients with type 1 and type 2 diabetes: a meta-analysis of observational studies

SUPPLEMENTARY MATERIALS

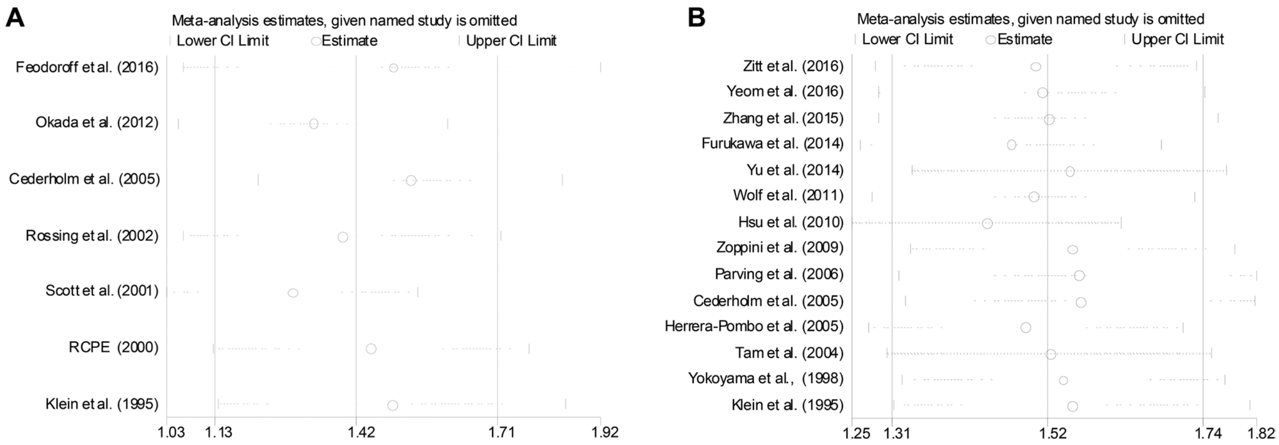

Supplementary Figure 1: Influence of removing studies one by one on the association between ever smoking and the development of diabetic nephropathy in patient with type 1 (**A**) and with type 2 diabetes (**B**). Circles are effect estimates and horizontal dotted lines were 95% confidence intervals for meta-analysis of the remained studies; the vertical line in the center is the pooled effect estimate for all studies.

Supplementary Table 1: Characteristics of observational studies of the association between smoking and diabetic nephropathy. See_ Supplementary_Table 1
